# Levels of scientific evidence of the quality of life in patients treated for oral cancer

**DOI:** 10.4317/medoral.19052

**Published:** 2013-05-31

**Authors:** Rocío Barrios, Javier Montero, Miguel A. González-Moles, Pilar Baca, Manuel Bravo

**Affiliations:** 1 DDs, Postgraduate Research Fellow of the Spanish Ministry of Education. School of Dentistry. University of Granada; 2 PhD, Associate Professor of Prothetic Dentistry. School of Medicine. University of Salamanca; 3 PhD, Professor of Oral Medicine. School of Dentistry. University of Granada; 4 PhD, Professor of Preventive and Community Dentistry. School of Dentistry. University of Granada

## Abstract

Treatments used in cancer of the oral cavity have great impact on the physical, psychological and functional state of patients. There has been increasingly interest in evaluating the health-related quality of life using questionnaires among patients treated FOR oral cancer. Up to our knowledge no review on this theme has incorporated the level of evidence of the single identified studies. The objective of the present study is to determinate results and conclusions about the health-related quality of life of these patients, in view of scientific evidence. In general, the diversity of designs, level of evidence and questionnaires used for their assessment does not affect results, which indicate a decline in the health-related quality of life after treatment. This decline is greater when the tumor is large in size, and when radiotherapy is used, though the situation is seen to improve over the span of a year. Questionnaires on health-related quality of life provide concrete information regarding the impact of cancer treatment on patients.

** Key words:**Quality of life, oral cancer, questionnaire.

## Introduction

Cancer of the oral cavity is a pathology of increasing significance worldwide. The approximately 263,000 new cases every year make it the most common form of head/neck cancer; mortality rates indicate that over half of these cases survive (127,654 deaths per year) (1). Such figures indicate that a considerable number of patients cope with the after-effects of the treatment: surgery or the habitual combination of surgery, radiotherapy and chemotherapy.

A patient endures stressful situations from the diagnosis of oral cancer. In advanced stages, and after treatment, changes associated with chewing, swallowing, salivating and speech are seen (2,3). Thus, the patient´s health-related quality of life (HRQoL) may be altered considerably.

For these reasons, there is growing interest in evaluating the HRQoL using questionnaires among patients treated for oral cancer (4,5). The patient completed questionnaires are the most common method used to assess the HRQoL (2). They are composed for questions or items whose answers can be analyzed independently and be combined to obtain different domains (6).

The development of a hierarchy in the scientific evidence has allowed health professionals to do an evidence-based clinical practice (7). Up to our knowledge no review on HRQoL in patients treated FOR oral cancer has incorporated the level of evidence of the single identified studies. The aim of our study was to determinate results/conclusions about HRQoL drawn by studies of patients treated for oral cancer, and to identify possible differences according to the study design or level of scientific evidence.

## Material and Methods

A bibliographic search was done using MEDLINE and Scopus databases, with the strategy (“oral cancer” OR “mouth neoplasms”) AND “quality of life” AND “questionnaire” in the period 1966-Dic 2012. Quality of life was used as the main search term, as it is a more common keyword than HRQoL (8). Furthermore, studies included in the bibliographic references of these papers were identified. After this initial search, the abstracts were read and the full paper if necessary, in light of the inclusion criteria in this study. The articles were included if they assessed the HRQoL, psychological aspects and/or functional aspects of patients treated for cancer of the oral cavity, oral and lip cancer, and/or oropharyngeal cancer, using a HRQoL questionnaire. The studies that incorporated other head neck cancers (nasal cavity, nasopharynx, larynx…) were excluded. This process resulted in 79 articles, referring to 62 independent studies.

After that, the documents were classified according to their design as.

-Randomized clinical trials: randomized intervention studies where, after applying different treatments in cancer patients, the results are compared using HRQoL questionnaires.

-Studies of controlled cohorts: those evaluating the quality of life of the patients on one occasion (without repeated measurements) or on numerous occasions (longitudinal study), the results being compared with those of a control cohort or with populational data existing in the literature.

-Studies of non-controlled cohorts: longitudinal studies whose results were not contrasted with any other cohort.

-Transversal studies: those taking one measurement of the quality of life of patients without comparing results with any other cohort.

-Reviews: articles that offer an updated overview of the findings of previous studies.

Furthermore, the designs were coded according to the following levels of scientific evidence (9): 1 ++) Systematic review or meta-analysis of randomized clinical studies of a high quality or randomized clinical studies with a very low risk of bias. 1 +) Systematic review or meta-analysis of randomized clinical trials that were well conducted or randomized clinical trials with a low risk of bias. 1 –) Systematic review or meta-analysis of randomized clinical trials, or randomized clinical trials with a high risk of bias. 2 ++) Systematic review of cohort studies or case controls of high quality, or systematic review of cohort studies or case controls of high quality and entailing a low risk of confounding factors, bias or chance, and a high probability of causal association. 2 +) Studies of cohorts or case controls that were well conducted, with a low level of confounding factors, bias, chance and a moderate probability of causal association. 2 –) Studies of cohorts or case controls with a high level of confounding factors, bias, chance and a significant risk that the association was not causal. 3) Non-analytical studies, such as cross-sectional surveys or case series. 4) Expert opinions or non-systematic reviews.

No article selected for our study was excluded for methodological reasons (10,11). The authors summed up the main results and conclusions from the articles included under the different types of study designs. To achieve final consensus, a focal group was organized consisting of the authors of this article and following the EuropeAid Evaluation Guidelines (12). Briefly, a focal group is a qualitative research technique that involves moderated meetings in the form of a structured, open group interview. After exposition and discussion of the different opinions, a final report was written up with the results and conclusions agreed upon.

## Results

T Table 1 shows the results of the bibliographic search conducted. There was an increase in the number of publications over time. We found that the general questionnaires were used in most often (European Organization for Research and Treatment of Cancer Quality of Life Questionnaires, EORTC-QLQ, closely followed by University of Washington Quality of Life Questionnaire, UW-QOL). Only four studies used the oral health impact profile (OHIP), a specific questionnaire for assessing changes in the oral health-related quality of life.

**Table 1:**
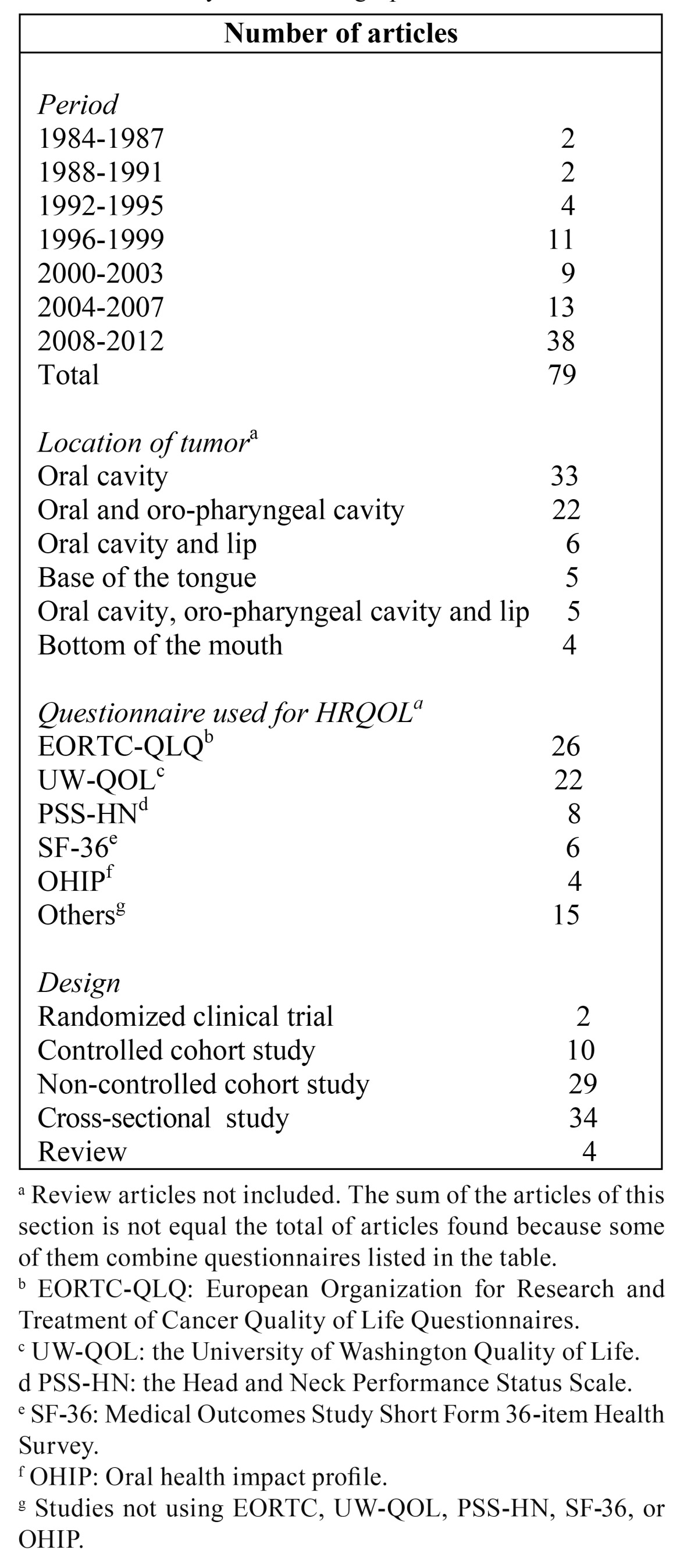
Summary of the bibliographic search.

The main results and conclusions from the articles are presented according to their design in the  Table 2. Only 2 experimental studies were found: one study classified as 1 ++ (maximum level of evidence), and another with a level 1 +. There were 10 con-trolled cohort studies, with a 2 + level of evidence, whose results were not conclusive. The 29 studies of non-controlled cohorts together with the 34 cross-sectional studies (level of scientific evidence 3) plus the 4 reviews found (level of evidence 4) make manifest that the patients treated with radiotherapy have a poorer HRQoL.

**Table 2:**
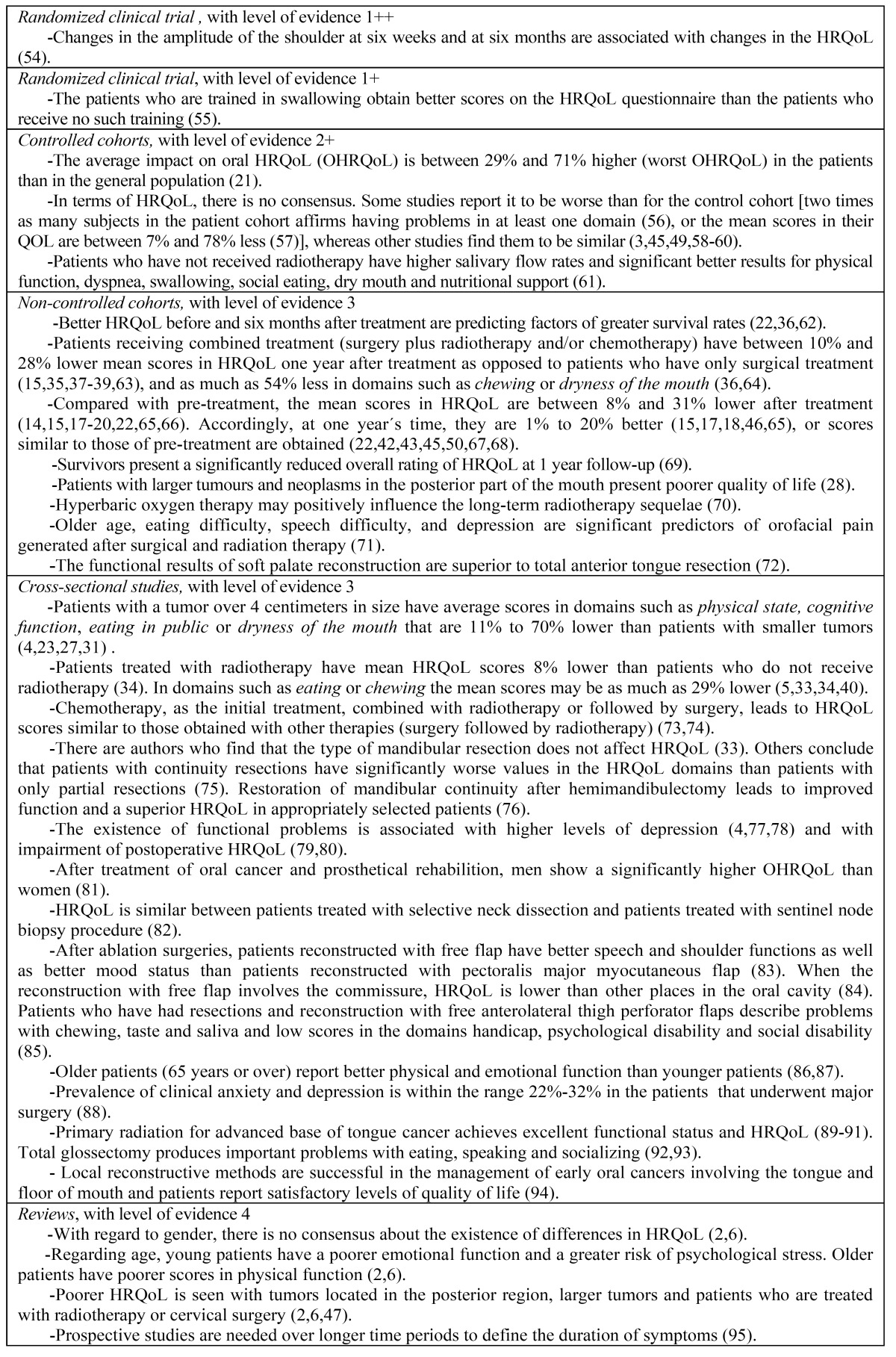
Summary of main findings/conclusions from studies, according to study design and level of evidence.

## Discussion

Quality of life has been considered to be an important outcome parameter in oral cancer (8), which explains the increasing number of studies on HRQoL found over the time period analyzed here. There is great diversity in the questionnaires used, or even combined, perhaps due to the fact that the concept of HRQoL is associated with multiple functional and psychosocial factors. The existence of a gold standard with unified criteria would allow the studies to be compared in a more rigorous manner. Overall, however, they do manage to cover the most common problems in a structured way, proving to be a useful tool for enhancing communication between health professionals and patients, which is essential in the area of cancer (6,13).

Despite the heterogeneity of the designs, the results/conclusions are on the same line: a decrease in the HRQoL after treatment (14-22), which appears to parallel the magnitude of the tumor (2,4,23-31), and the use of radiotherapy (2,4,5,29,32-40). After a year, this declining trend turns around (15,16,18,20,22,41-47). The improvement in HRQoL over time is a result to be taken with some caution. Coping mechanisms, or adaptation to a new situation, may be one reason, but we must not forget that the data are based on surviving patients without relapse.

The existence of two randomized clinical trials evidences the practicality of incorporating the measure of HRQoL in these types of studies. Accordingly, HRQOL would have sufficient scientific backing to become a key consideration in the treatment planning process, as in situations where there are virtually no differences in associated survival rates (48). Moreover, we should underline the lack of studies with controlled cohorts. The most of them compare the results of the patients with populational data or with the results of their partners/spouses whose life quality is likewise affected by the illness (3,49). It would be interesting to carry out new research studies designed in such a fashion that each patient would have a control paired up by age and sex that would allow for comparison of results in a less biased way.

Despite therapeutic advances and enhanced survival, oral cancer patients inevitably face some decrease in HRQoL (28,50). They are not always satisfied with the information received, especially in relation to the changes they experience in their lifestyle after treatment(51,52). Health professionals have a variety of validated questionnaires, allowing them to familiarize themselves with the after-effects of oral cancer and therapy to the improvement of this communication. Online questionnaires can be used to overcome the lack of time and resources of health professionals dealing with these patients (53).

In conclusion, the diversity of study designs (level of evidence) does not appear to affect the results of studies. Construction of a standard questionnaire and its use in studies with a high scientific level of evidence would help make the differences found in the HRQoL become an important element in planning treatment for patients with oral cancer.
